# Frequency shifts in the anterior default mode network and the salience network in chronic pain disorder

**DOI:** 10.1186/1471-244X-13-84

**Published:** 2013-03-13

**Authors:** Alexander Otti, Harald Guendel, Afra Wohlschläger, Claus Zimmer, Michael Noll-Hussong

**Affiliations:** 1 Klinik und Poliklinik fuer Psychosomatische Medizin und Psychotherapie, Klinikum rechts der Isar, Technische Universitaet Muenchen, Langerstrasse 3, Muenchen, D-81675, Germany; 2 Abteilung fuer Neuroradiologie, Klinikum rechts der Isar, Technische Universitaet Muenchen, Ismaningerstrasse 22, Muenchen, D-81675, Germany; 3 Klinik und Poliklinik fuer Psychosomatische Medizin und Psychotherapie, University of Ulm, Albert-Einstein-Allee 23, Ulm, D-89081, Germany

**Keywords:** Chronic pain disorder, Somatoform pain disorder, Resting state networks, Intrinsic connectivity networks, Functional brain imaging, fMRI

## Abstract

**Background:**

Recent functional imaging studies on chronic pain of various organic etiologies have shown significant alterations in both the spatial and the temporal dimensions of the functional connectivity of the human brain in its resting state. However, it remains unclear whether similar changes in intrinsic connectivity networks (ICNs) also occur in patients with chronic pain disorder, defined as persistent, medically unexplained pain.

**Methods:**

We compared 21 patients who suffered from chronic pain disorder with 19 age- and gender-matched controls using 3T-fMRI. All neuroimaging data were analyzed using both independent component analysis (ICA) and power spectra analysis.

**Results:**

In patients suffering from chronic pain disorder, the fronto-insular ‘salience’ network (FIN) and the anterior default mode network (aDMN) predominantly oscillated at higher frequencies (0.20 - 0.24 Hz), whereas no significant differences were observed in the posterior DMN (pDMN) and the sensorimotor network (SMN).

**Conclusions:**

Our results indicate that chronic pain disorder may be a self-sustaining and endogenous mental process that affects temporal organization in terms of a frequency shift in the rhythmical dynamics of cortical networks associated with emotional homeostasis and introspection.

## Background

Chronic pain disorder, as defined in the DSM-IV [1], is a somatoform disorder lasting longer than 6 months in which the predominant symptoms are bodily complaints of pain. Psychological factors are thought to be central to the onset, severity, exacerbation and maintenance of the complaint. Characteristically, patients with this clinically prevalent disorder have difficulties recognizing and interpreting emotional signals within themselves; they perceive these signals as physical symptoms [2]. Moreover, the disorder itself leads to significant neural alterations in regions associated with emotional awareness [3], affective meaning construction [4], and bodily state monitoring [5], such as the medial prefrontal cortex, the anterior cingulate cortex, and the insula [6].

In addition to studies concerning morphology and paradigm-based activations, the temporal dimension of neural processing has recently gained attention [7-9]. This dynamic view of brain functioning emphasizes the importance of the functional interplay between different brain regions, with a particular focus placed on altered resting state connectivity in mental disorders [10]. One of the strongest disruptors of this complex equilibrium seems to be pain [11-14]. In a recent study of 10 patients suffering from nociceptive chronic pain, the spatial coherence of the fronto-insular ‘salience’ network (FIN) was altered in the resting state [15]. Chronic pain influenced the temporal aspects of functional connectivity by changing the frequency of the rhythmic oscillations in the BOLD-signal within the FIN from lower levels (below 0.12 Hz) to a higher range (between 0.12 and 0.24 Hz) [15]. Moreover, chronic back pain seems to disrupt the integrity of the so-called default mode network (DMN) [11], whereas diabetic neuropathic pain changes the temporal coherence of the DMN [16].

Interestingly, chronic pain not only influences neural circuits but also tends to operate in a domain-general manner. Neuropathic diabetic pain, for example, also changes the spatial functional anatomy of the sensorimotor network (SMN) [16]. However, the aforementioned studies [15,16] have focused on chronic pain conditions without distinguishing between pain that can be clearly associated with a convincing organic correlate and somatoform pain (e.g., in chronic lower back pain [17]) or generalized pain.

Thus, the present study aims to fill this gap, examining whether chronic pain disorder patients show similar alterations in frequency and functional connectivity within the brain’s functional architecture. We define chronic pain disorder as pain that is not the result of a clear organic etiology or that is out of proportion to the intensity of physical findings and that is caused by a well-classified mental disorder (ICD-10: F45.4x, DSM-IVR: 307.80), characterized predominantly by chronic ongoing pain [1,18]. Given that there is an endogenous central process that is observed in chronic pain disorder, we hypothesize that pain-related resting state networks such as the DMN, FIN, and SMN will fluctuate at even higher frequencies in patients than in healthy controls. We also hypothesize that these networks will show evidence of disturbed spatial functional connectivity.

## Methods

This study was approved by an institutional ethics committee (Klinikum rechts der Isar, Medical Faculty of Technische Universitaet Muenchen, Germany) and was performed in accordance with the Declaration of Helsinki.

Nineteen healthy controls (mean age: 48.79 years, SD 12.25, 12 females) and 21 German-speaking patients (mean age: 46.62 years, SD 12.49, 17 females) with chronic pain disorder, defined as a pain-predominant multisomatoform disorder diagnosed by an experienced physician using a modified SCID-I interview, provided informed written consent and participated in the experiment. The main feature of somatoform disorders is “the repeated presentation of physical symptoms together with persistent requests for medical investigations, despite repeated negative findings and reassurances by physicians that the symptoms have no physical basis. If any physical disorders are present, they do not explain the nature and extent of the symptoms or the distress and preoccupation that the patient has with them” [18]. Multisomatoform disorder, a medium-to-severe somatoform disorder, is defined as three or more medically unexplained, currently bothersome, physical symptoms in addition to a long (≥ 2 years) history of somatization [19]. Because of the striking comorbidity of multisomatoform disorder with major depression and anxiety disorders, it has been suggested that overlapping psychobiological mechanisms mediate depression, anxiety, and somatization symptoms [20]. Compared with mood and anxiety disorders alone, multisomatoform disorder is associated with comparable impairments in health-related quality of life, a greater number of self-reported disability days and clinic visits, and the highest levels of provider frustration [21,22].

The Physical Component Summary (PCS) measure [23] in our patient group had to be 1 standard deviation or more below the population norm (≤ 40), as measured with the SF-36 (see below). A score less than 40 also meets the DSM-IV criterion B for “significant distress or psychosocial impairment due to the somatoform pain” in patients with pain disorder [1]. As a second precondition, sum scores on the 15-item Patient Health-Questionnaire (PHQ-15) had to be above 10, representing at least medium somatic symptom severity (see below). The German version of the Brief Pain Inventory (BPI) [24] was used to estimate the intensity of each participant’s pain. We reviewed patients’ medical charts and contacted the treating physicians to rule out possible or unclear organic explanations for the symptoms of our chronic pain patients. Patients with insufficient cognitive abilities, severe and chronic somatic or nervous diseases, unambiguous nociceptive pain, hypochondriasis, a severe comorbid mental disorder causing major impairment in social functioning (e.g., schizophrenia or severe substance abuse) or insufficient German language skills were excluded. All participants were white, of Caucasian origin, and right handed, as assessed by the Edinburgh handedness inventory [25]. Additional file 1: Table S6 lists all medications that patients were currently taking.

### Psychometric measurement

Somatoform disorders were diagnosed using a modified semi-structured psychiatric interview, the German version of the SCID-I (Structured Clinical Interview for DSM Disorders) [26]. The SCID-I is the diagnostic criterion standard and evaluates current (i.e., the 4 weeks preceding the interview) and lifetime psychiatric status for major Axis I mental disorders using criteria that correspond to the DSM-IV [1].

The SF-36 is a multipurpose, short form health survey consisting of 36 questions [27]. It yields an 8-scale profile of functional health and well-being scores, psychometrically based physical and mental health summary measures, and a preference-based health utility index. It is a generic measure, as opposed to one that targets a specific age, disease, or treatment group. Accordingly, the SF-36 has proved useful in surveys of both general and specific population groups. It compares the relative burden of disease and differentiates the health benefits generated by a wide range of different treatments [28]. Its German translation has been validated in a variety of German health care settings [29,30].

The PHQ-15 is a brief, self-administered questionnaire that has proved useful in screening for somatization and in monitoring somatic symptom severity in clinical practice and in research. Scores of 5, 10, and 15 represent the cutoff points for low, medium, and high somatic symptom severity, respectively [31,32].

The BPI, based on the Wisconsin Brief Pain Questionnaire, was developed by the Pain Research Group of the WHO Collaborating Centre for Symptom Evaluation in Cancer Care to provide information on the intensity of pain (the sensory dimension) and the degree to which pain interferes with function (the reactive dimension) [33]. The validity of the German version [24] and the ability of the BPI to measure pain in patients without cancer [34] have been demonstrated.

The applied Beck Depression Inventory I (BDI-I) is a 21-item self-reported instrument that measures cognitive and endogenous aspects of depression on a four-point scale ranging from 0 to 3. The standard cut-offs are as follows: 0–9 indicates no depression, 10–18 indicates mild depression, 19–29 indicates moderate depression, and >30 indicates severe depression. This questionnaire has undergone extensive reliability and validation studies [35,36].

The German version of the Trait Anxiety Inventory (STAI-T) is a valid and reliable 20-item questionnaire that measures the general level of anxiety on four-point scales ranging from 1 to 4 [37].

### Functional MRI resting state paradigm

Participants were asked to close their eyes and relax but to remain awake. This portion of the experiment lasted 370 seconds. Following the scanning session, participants were asked whether they had fallen asleep during the scan; those who provided a positive or ambiguous answer were excluded from the study.

### Data acquisition and fMRI procedures

Images were acquired with a 3T Philips Achieva Scanner (Philips Medical Systems, Best, The Netherlands) using a standard 8-channel SENSE head coil. Thirty-two contiguous slices (no gap), with a steep angulation to exclude the eyes, were acquired using a gradient echo-planar (EPI) sequence with the following parameters: 2000 ms repetition time (TR); 35 ms echo time (TE); 82 degree flip angle; 220 mm FOV; 4 mm slice thickness; 80_80 matrix; voxel size 2.75_2.75 mm; SENSE factor 2. Anatomical images were obtained using a T1-weighted turbo gradient echo sequence with the following parameters: 9 ms TR; 4 ms TE; 8 degree flip angle; 240 mm field of view (FOV); 240_240 matrix; voxel size 1 mm isotrop; 170 slices; no gap.

### Data analysis and image processing

Data analysis was performed using SPM5 (Statistical Parametric Mapping software, Wellcome Department of Imaging Neuroscience, London, UK; http://www.fil.ion.ucl.ac.uk). The first three images for each run were discarded to allow for equilibration of longitudinal magnetization. The preprocessing steps included (1) realignment and unwarping of the images to correct for movement artifacts and related susceptibility artifacts, (2) coregistration of the anatomical images to the functional images, (3) segmentation and normalization of the anatomical images to a standard stereotactic space (Montreal Neurological Institute, MNI; Quebec, Canada), (4) application of a normalization transformation to the functional images, and (5) smoothing with a Gaussian kernel of 8 mm for group analysis.

### Connectivity analysis

We performed an independent component analysis (ICA) by using the “group ICA” function included in the fMRI toolbox (*GIFT version 1.3h*; http://icatb.sourceforge.net) developed for the analysis of fMRI data [38-40]. First, the individual data were concatenated across time, followed by the computation of subject-specific components and time courses. The analysis proceeded in three stages: (1) data reduction, (2) application of the ICA algorithm, and (3) back reconstruction for each individual subject [38]. In the first step (1), data from each subject underwent principal component analysis to reduce the computational complexity of the analysis. In so doing, most of the content of the data was preserved. After concatenating the resulting volumes, the number of independent sources was estimated using the GIFT dimensionality estimation tool based on the aggregated data and using the minimum-description-length criteria [41]. The final reduction step, according to the selected number of components, was achieved again using principal component analysis. In the second stage of the analysis (2), we used the *Infomax* algorithm to run the appropriate ICA and a mask based on all subjects. In the final stage of back reconstruction (3), time courses and spatial maps were computed for each subject. The resulting mean spatial maps of each group were transformed to z scores for display [38].

Individual subject maps of the ICNs were entered into random effects analyses in SPM5. The results were thresholded at p = 0.05 and corrected for family wise error (FWE) with a cluster extent threshold of 50 voxels.

To enhance both the reliability and validity of this study, the ICNs were compared with networks that were calculated from a sample of approximately 600 healthy people in a study previously published by Allen et al. [42] that used spatial correlation (multiple regression) in the GIFT program [38] (see below for details).

For comparison between groups, we used two-sample t-tests with the available psychometric depression and anxiety scores as covariates of no interest. To detect even weak effects, a more lenient threshold was used for the group comparison (p = 0.005, uncorrected on the voxel level (z > 2.58), and p = 0.05, corrected for multiple comparisons on the cluster level, extent threshold k > 10 voxels). Correlation analysis was performed at the same threshold. The connectivity maps from GIFT were entered into SPM5. We performed a partial correlation analysis (Pearson correlation) between functional connectivity and the level of depression on the BDI-I, controlling for the level of anxiety on the STAI-T. We also performed a partial correlation analysis between functional connectivity and the level of anxiety on the STAI-T, controlling for the level of depression on the BDI-I. Finally, we correlated the average subjective pain during the last week (item 5 on the BPI) with the functional connectivity using a bivariate correlation.

### Power spectra analysis

The GIFT toolbox “spectral group compare” function was used to calculate power density frequency spectra for each subject at six equally spaced frequency bins between 0 and 0.24 Hz at 0.04 Hz intervals (2-sample t-test, p < 0.0083 ≅ 0.05/6; Bonferroni-correction for 6 frequency bins). Several previous studies have also used power-spectra analysis (see [15,16,43,44]; please note that the number of bins and the intervals are different in each study). The level of depression (BDI-I) and the level of anxiety (STAI-T) were introduced as nuisance covariates. Correlation analyses with all psychometric data were performed at the same threshold.

## Results

### Pain ratings

Prior to scanning, the German version of the Brief Pain Inventory (BPI) was used to estimate the intensity of the patients’ chronic pain during the previous week. On average, subjects rated their pain as a 7 (SD 2.24) using a Numerical Rating Scale (NRS), which ranged from 0 (*“no pain”*) to 10 (*“pain as bad as you can imagine”*) on item 5 of the BPI. For comparison, in cancer-induced bone pain, the most common cause of pain in patients with cancer, the median average pain using the BPI was found to be 4 [45]. All patients suffering from chronic pain disorder experienced pain throughout the fMRI scan.

### Psychometric measurement

Patients with chronic pain disorder showed significantly higher BDI-I levels in the form of mild depression, higher trait-anxiety (STAI-T) scores and higher pain levels on the BPI (item 5) compared with the control group (Table 1). The level of depression was significantly correlated with the level of anxiety (R = 0.593, p = 0.005). No relevant correlation was observed between the level of clinical pain (BPI, item 5) and the level of depression (R = - 0.01, p = 0.996) or the level of anxiety (R = 0.083, p = 0.736).

**Table 1:** Averages and comparisons of group scores

	**Patients**				**Controls**				**t-Test -p-value;**
	**Mean**	**Median**	**SD**	**Range**	**Mean**	**SD**	**Median**	**Range**	
**BPI (Item 5)**	7	6	2.24	2 - 9	0	0	0	-	0.000
**BDI-I:**	17.84	20	9.03	3 - 37	4.43	4.70	2	0 -16	0.000
**STAI-T**	47.10	49	12.4	20 -70	35.94	8.56	34	23 - 50	0.002

Two-sample t-tests of average pain intensity (BPI), depression (BDI-I) and trait-anxiety (STAI-T) in patients with chronic pain disorder and healthy controls. The threshold of significance is p < 0.05.

### Functional MRI data – spatial connectivity analysis (Figures 1 and 2)

**Figure 1:**
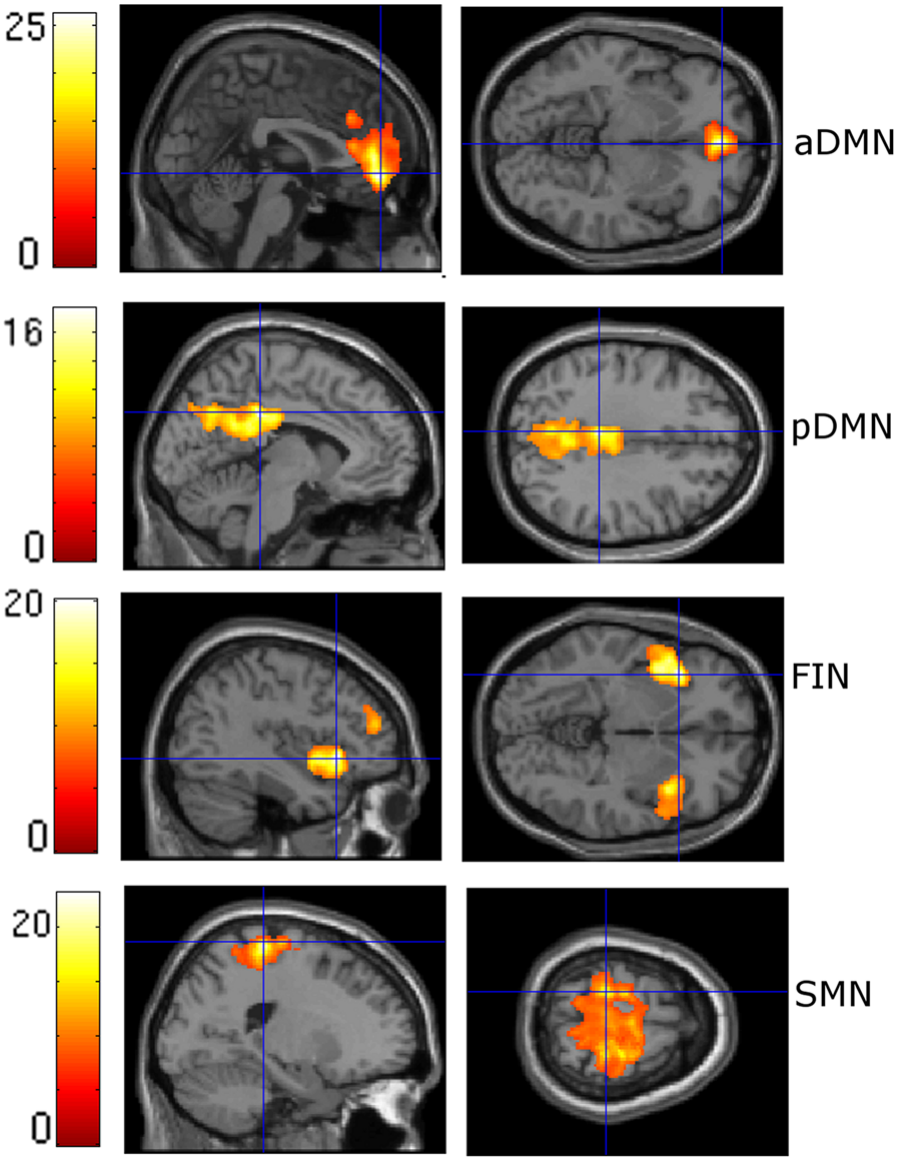
**ICNs of the control group.** For illustration purposes, spatial maps were thresholded at P = 0.05, corrected for family wise error (FWE) with a cluster extent threshold of 50 voxels; aDMN = anterior default mode network, pDMN = posterior default mode network, FIN = fronto-insular network, SMN = sensorimotor network.

**Figure 2:**
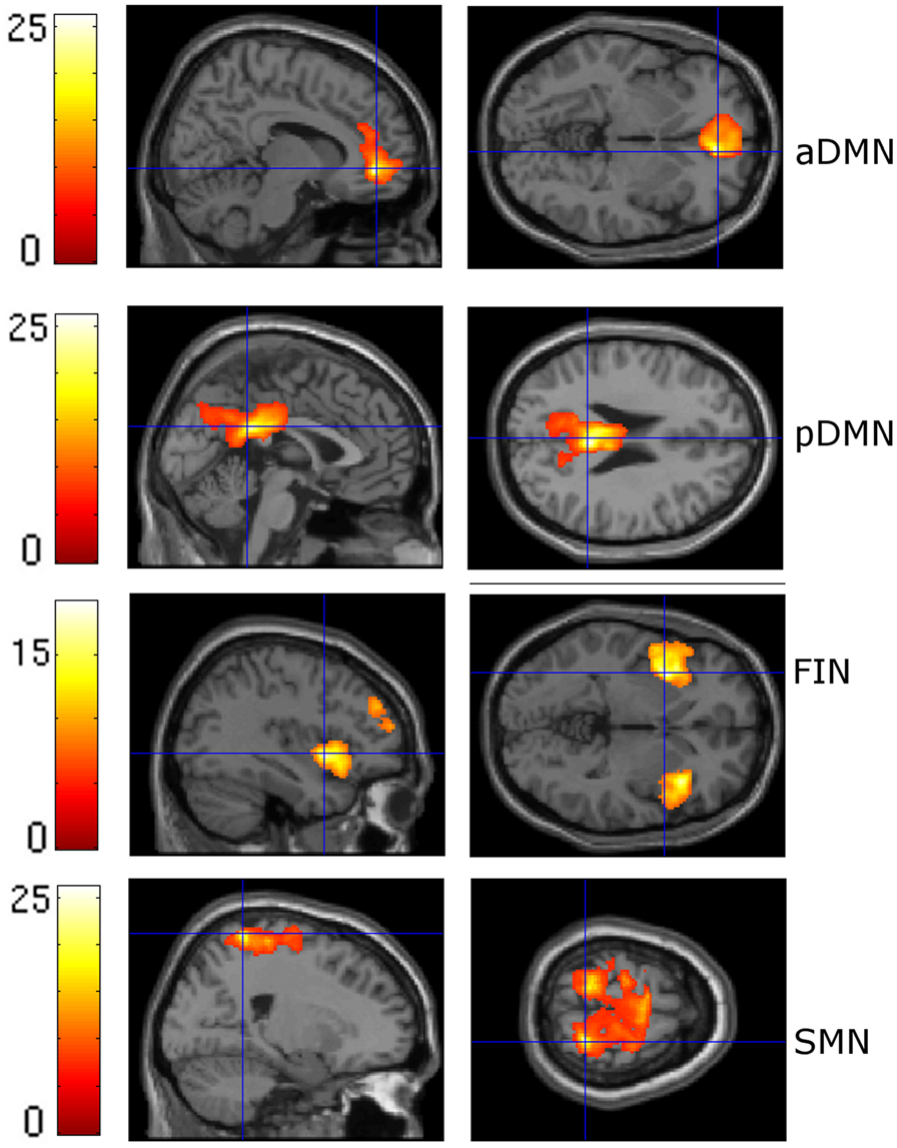
**ICNs of the patient group.** For illustration purposes, spatial maps were thresholded at P = 0.05, corrected for family wise error (FWE) with a cluster extent threshold of 50 voxels; aDMN = anterior default mode network, pDMN = posterior default mode network, FIN = fronto-insular network, SMN = sensorimotor network.

The ICA estimation resulted in 29 independent components. In accord with published data from other groups, we identified the following pain-related networks (Figures 1 and 2, Additional file 2: Table S1, Additional file 3: Table S2):

1. The anterior default mode network (aDMN), which comprises cortical midline structures such as the medial prefrontal cortex and the precuneus [11,12,16,46]. The aDMN showed the strongest overlap with component 25 from Allen et al. [42], which represents the anterior part of the default mode network (multiple regression value: 0.22).

2. The posterior default mode network (pDMN) of the precuneus [11,12,16,46]. The pDMN showed the strongest overlap with component 50 from Allen et al. [42], which represents the posterior part of the default mode network (multiple regression value: 0.14).

3. The fronto-insular network (FIN), which comprises both the insula and the cingulate cortex [15,47]. Component 55 from Allen et al. [42], which represents the fronto-insular salience network, showed the strongest overlap with this network (multiple regression value: 0.22).

4. The sensorimotor network (SMN), which comprises the pre- and post-central gyrus [48]. The SMN showed the strongest overlap with component 29 from Allen et al. [42], which represents a sensorimotor network (multiple regression value: 0.14).

No significant differences in spatial functional connectivity between the patient and control groups were detected (Additional file 4: Table S3). Moreover, no significant correlation was observed between the psychometrically measured level of pain (BPI), anxiety (STAI-T), depression (BDI-I) and spatial functional connectivity [42] in the patient group (Additional file 5: Table S4).

### Functional MRI data – power spectra analysis (Table 2, Figure 3)

**Table 2:** Comparison of power spectra for all ICNs between patients and healthy controls

**ICN**	**Group**	**Spectral power at different frequency-bins in percent of the whole power**					
		**0.0 – 0.04 Hz**	**0.04 – 0.08 Hz**	**0.08 – 0.12 Hz**	**0.12 – 0.16 Hz**	**0.16 – 0.20 Hz**	**0.20 – 0.24 Hz**
**aDMN**	**Controls**	31.732	20.831	12.677	15.703	12.415	**9.881**
	**Patients**	29.507	19.989	12.833	12.960	11.932	**15.351**
	**p-value (t-test)**	0.338	0.510	0.856	0.015	0.693	**0.001**
**pDMN**	**Controls**	29.651	22.137	13.550	16.374	12.520	9.312
	**Patients**	29.637	21.374	14.290	14.306	11.008	12.377
	**p-value (t-test)**	0.993	0.580	0.373	0.118	0.175	0.019
**FIN**	**Controls**	33.751	22.393	12.880	14.318	10.797	**9.067**
	**Patients**	31.438	22.477	13.702	12.661	9.854	**12.728**
	**p-value (t-test)**	0.262	0.933	0.260	0.179	0.378	**0.005**
**SMN**	**Controls**	36.671	19.570	14.069	13.729	10.771	7.827
	**Patients**	31.919	21.600	14.297	14.030	9.650	11.512
	**p-value (t-test)**	0.117	0.153	0.852	0.839	0.343	0.016

Two-tailed t-test, p < 0.05/6, significant differences are included in bold.

**Figure 3:**
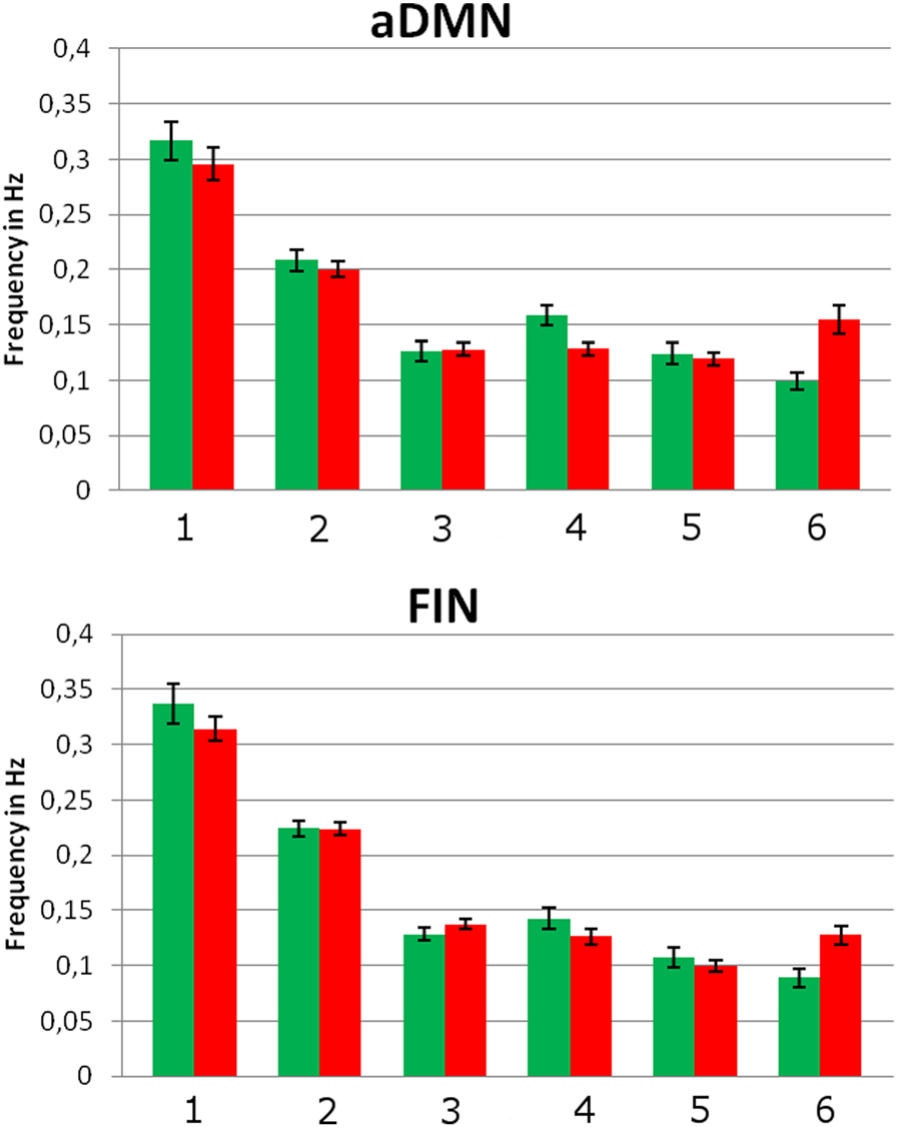
**Power spectra of patients (red) and healthy controls (green).** Intrinsic neural activity within the aDMN and the FIN show faster spontaneous fluctuations in patients with chronic pain disorder. Error bars represent the standard error of the mean. [1 ≡ 0–0.04 Hz, 2 ≡ 0.04 - 0.08 Hz, 3 ≡ 0.08 - 0.12 Hz, 4 ≡ 0.12 - 0.16 Hz, 5 ≡ 0.16 - 0.20 Hz, 6 ≡ 0.20 - 0.24 Hz].

Compared to the control group, patients showed higher power spectra in the aDMN and the FIN, ranging between 0.20 and 0.24 Hz. No significant correlation was observed among the level of pain, depression, trait-anxiety and spectral power (Additional file 6: Table S5). These group differences were not influenced by levels of depression and trait-anxiety as measured by the BDI-I and STAI-T, respectively.

## Discussion

This study reveals that neural activity within the FIN and the aDMN in patients with chronic pain disorder shows significantly shifted frequencies in comparison with healthy controls. Moreover, a general trend toward higher power in the 0.20 - 0.24 Hz frequency bin was evident in patients compared with control subjects. However, significant changes in the spatial dimensions of functional connectivity were not detected.

Our results support the study hypothesis that there is a shift of the endogenous oscillations of the brain’s resting state to higher frequencies in patients suffering from chronic ongoing pain, even when a physical examination cannot (fully) explain the subjective symptoms and the patients fulfill the official criteria for chronic pain disorder.

Furthermore, by demonstrating higher BOLD fluctuations in the FIN and DMN in chronic pain disorder, our findings expand the results of both Malinen et al. [15] and Cauda et al. [16]. Other authors have discovered similar alterations in temporal coherence among patients suffering from chronic neuropathic pain associated with obvious organic diseases [49,50]. Compared to previous studies on the brain’s temporal dynamics in chronic pain, we used a different binning strategy for spectral analyses. Malinen et al. [15] calculated spectral power at three frequency bins (0–0.05 Hz; 0.05 - 0.12 Hz; 0.12 - 0.25 Hz), whereas Cauda et al. [16] defined four intervals of interest (0.008 - 0.02 Hz; 0.02 - 0.05 Hz; 0.05 - 0.1 Hz; 0.1 - 0.25 Hz). In our study, six equally spaced frequency bins were used (0–0.04 Hz; 0.04 - 0.08 Hz; 0.08 - 0.12 Hz; 0.12 - 0.16 Hz; 0.16 - 0.20 Hz; 0.20 - 0.24 Hz). The main advantage of using 6 bins compared to a greater number of bins is that it reduces the number of multiple comparisons (level of significance p < 0.0083 ≅ 0.05/6; Bonferroni-correction for 6 frequency bins). A lower number of bins, however, might have led to false-negative results because the spectral changes are rapid, increasing as a function of frequency. Furthermore, whereas Malinen et al. [15] used a relatively broad interval for the higher frequencies (0.12 – 0.25 Hz), we were able to show that the upper end of the high-frequency interval (between 0.20 and 0.24 Hz), in particular, might be relevant in chronic pain disorder.

There was no significant correlation between shifts in frequency of the BOLD-signal and the psychometric level of anxiety [51], depression [20,52,53] or pain intensity in the patient group of our study. Nevertheless, we cannot definitely exclude the possibility that changes were not due to persistent somatoform pain but were due to other unknown variables. Furthermore, there was no significant correlation between spectral power and anxiety [51] or depression [20,52,53] Importantly, a similar discrepancy between BOLD activations and behavioral measurements was also described in a study investigating an altered cerebral response to noxious heat stimulation in patients with somatoform pain disorder [6]. Thus, differences between our two groups may be more easily detected via neuroimaging methods than through subjective behavioral ratings, in accord with several other studies [54-57].

Although our study does not demonstrate causal relationships, several findings suggest a strong relationship between pain-condition and altered spectral power. Somatoform pain is associated with higher autonomic arousal [58,59], which, in turn, has been associated with increased activation in the fronto-insular regions [16,60]. Although autonomic activation was not measured directly in our study, an altered psycho-vegetative state [57] might be the behavioral equivalent of increased FIN oscillations in chronic pain disorder, as proposed by Malinen et al. [15]. Remarkably, the FIN and DMN networks seem to be involved in affective neuroprocessing: Whereas the DMN subserves introspection, autobiographic memory, self-referential processing, and social understanding [61-64], the FIN has been linked with personal salience, emotional awareness, and bodily state monitoring [5,47,65]. Moreover, the various bodily complaints in patients with somatoform pain have consistently been associated with a high affective component of individual pain, which indicates impaired emotional regulation [66-69]. Given these data, one might synoptically speculate that our findings reflect one neurobiological facet of the strong clinical impression that patients who suffer from chronic pain disorder often show impaired subjective emotional awareness, affective meaning construction [4] and social understanding [3].

No significant group differences were detected in the SMN, although previous studies have shown that chronic pain leads to functional reorganization, decreased gray matter density, and increased metabolism within the somatosensory cortex [70-74]. One might speculate that chronic pain disorder relies more on disturbed affective and introspective processing than on the disturbed somatosensory circuits that occur in patients who suffer from pain dependent on nociceptive input, for example, in a patient with posttraumatic osteoarthritis in the sample in Malinen et al. [15].

We did not find changes in spatial functional connectivity, in contrast to Malinen et al. [15], who reported weaker functional connectivity between the insula and anterior cingulate cortex in predominantly nociceptive chronic pain, and Baliki et al. [11], who found diminished DMN-connectivity in chronic back pain patients. In contrast to pain caused by diverse peripheral causes, we presume that chronic somatoform pain, which at least cannot be fully explained by possible nociceptive input, is not associated with alterations in the spatial and functional architecture of the brain’s resting state.

Altogether, chronic pain disorder seems to be associated with a frequency shift in the anterior default mode network and the salience network to higher (eigen)frequencies. The resting state of the human brain is thought to serve as a ´memory of the future´ [63,75], which stores behavioral algorithms to allow a person to adequately cope with upcoming environmental events. Therefore, our research on resting state connectivity as a special form of neuronal oscillations in cortical networks [76] might provide a useful neurobiological framework that underlies one facet of the behavioral changes that impair the daily lives of patients with chronic pain disorder.

## Conclusions

Though our study does not ascribe causation, our results indicate that patients suffering from chronic pain disorder show distinct alterations in the temporal organization of their brains. A persistent peripheral algetic input does not seem to be pivotal for changes in the functional architecture of the human brain associated with persistent somatoform pain in patients with chronic pain disorder.

### Limitations

The present study is limited because of the lack of measurements of possible sources of physiological artifacts (e.g., respiration, cardiac function and blood pressure). However, high agreement with previous findings of alterations in temporal activity in the FIN and the DMN suggests that our results were most likely not confounded by these factors [15,16]. The analgesic and antidepressant medication administered to most of our outpatients (Additional file 1: Table S6) could have influenced the reported frequency shift [77,78]; the enduring influence of such drugs on BOLD oscillations is currently still unknown. It is noteworthy that, despite ethical reasons, it was nearly impossible to convince our patients with chronic pain disorder to interrupt their psychotropic medication in this intentionally naturalistic study.

## Competing interests

The authors declare that they have no competing interests.

## Authors’ contributions

MN-H designed and conducted the research, analyzed the data, and contributed to the writing of the paper. AO conducted the research, analyzed the data, and contributed to the writing of the paper. AMW designed and performed the research. CZ and HG designed the research. All authors discussed the results and commented on the manuscript. All authors read and approved the final manuscript.

## Pre-publication history

The pre-publication history for this paper can be accessed here:

http://www.biomedcentral.com/1471-244X/13/84/prepub

## Supplementary Material

Additional file 1: Table S6 Medication of all 21 patients with chronic pain disorder. Click here for file

Additional file 2: Table S1 MNI-coordinates of the ICNs in the control group. Results were thresholded at p = 0.05 and corrected for family wise error (FWE) on the voxel level with a cluster extent threshold of k = 50 voxels. Click here for file

Additional file 3: Table S2 MNI-coordinates of the ICNs in the patient group. Results were thresholded at p = 0.05 and corrected for family wise error (FWE) on the voxel level with a cluster extent threshold of k= 50 voxels. Click here for file

Additional file 4: Table S3 MNI-coordinates of the group comparisons. Results were thresholded at p = 0.005, uncorrected at the voxel-level, and p < 0.05, corrected for multiple comparisons on the cluster level, with a cluster extent threshold of k = 50 voxels; p represents p on the voxel-level. Click here for file

Additional file 5: Table S4 Correlation between functional connectivity and psychometric measurement. Results were thresholded at p < 0.005, uncorrected on the voxel-level, and p < 0.05, corrected on the cluster level, with a cluster extent threshold of k > 10 voxels; p represents p on the cluster level; R represents Pearson’s correlation-coefficient. No significant correlation was detected. Click here for file

Additional file 6: Table S5 Pearson’s correlation between spectral power and psychometric measurements *The correlation with depression (BDI-I) is controlled for anxiety (STAI-T) and vice versa; the level of significance is p < 0.05; R represents the correlation-coefficient. No significant correlation was detected*.* Click here for file
