# Supplementary material for: Frequency shifts in the anterior default mode network and the salience network in chronic pain disorder
Source: BMC Psychiatry. 2013 Mar 13;13:84. doi: 10.1186/1471-244X-13-84 (PMC3616999; doi:10.1186/1471-244X-13-84)
Supplement: Additional file 1: Table S6 — Medication of all 21 patients with chronic pain disorder. [file 1471-244X-13-84-S1.doc]

**Table S6** **Medication of all 21 patients with chronic pain disorder**

| *Patient* | *Drug(s)* |
| --- | --- |
| p01 | Oxycodone, Citalopram, Valsartan/Hydrochlorothiazide |
| p02 | Amitriptyline, Paroxetine |
| p03 | Ibuprofen, Hypericin |
| p04 | Oxycodone/Naloxone, Pregabalin, Amitriptyline, Tramadol, Tetrazepam, Omeprazole, Lynestrenol |
| p05 |  |
| p06 |  |
| p07 | Oxcarbazepine |
| p08 | Hypericin; Cimicifuga racemosa |
| p09 | Tramadol, Amitriptyline |
| p10 |  |
| p11 | Pregabalin, Citalopram, L-Thyroxine |
| p12 |  |
| p13 | Metformin, Simvastatin, Pioglitazone |
| p14 | Diclofenac, Mirtazapine |
| p15 |  |
| p16 | Irbesartan |
| p17 | Tilidine/Naloxone, Pregabalin, Doxepin, Esomeprazole |
| p18 | Oxazepam |
| p19 | Pregabalin, Hypericin |
| p20 | Amitriptyline, Atenolol, Chlorthalidone |
| p21 | Amitriptylin, Novaminsulfone, Hydromorphone, L-Thyroxine, Lercanidipine, Atenolol, Rampril/Hydrochlorothiazide, Acetylsalicylic acid, Allopurinol, Simvastatin |
