# Supplementary material for: Frequency shifts in the anterior default mode network and the salience network in chronic pain disorder
Source: BMC Psychiatry. 2013 Mar 13;13:84. doi: 10.1186/1471-244X-13-84 (PMC3616999; doi:10.1186/1471-244X-13-84)
Supplement: Additional file 2: Table S1 — MNI-coordinates of the ICNs in the control group. Results were thresholded at p = 0.05 and corrected for family wise error (FWE) on the voxel level with a cluster extent threshold of k = 50 voxels. [file 1471-244X-13-84-S2.doc]

**Table S1 MNI-coordinates of the ICNs in the control group** Results were thresholded at p = 0.05 and corrected for family wise error (FWE) on the voxel level with a cluster extent threshold of k = 50 voxels.

| **Network** | **Region** | **MNI** | **k** | **T** | **p** |
| --- | --- | --- | --- | --- | --- |
| **aDMN** | R gyrus frontalis medius, pars orbitalis | 2 50 -6 | 2650 | 26,12 | 0.000. |
| **pDMN** | L middle cingulate cortex | -6 -34 34 | 2805 | 17.64 | 0.000. |
| **FIN** | L insula | -38 20 -4 | 1455 | 20.06 | 0.000. |
|  | R insula | 38 22 -16 | 1112 | 18,73 | 0.000. |
|  | L supplementary motor area | 0 10 64 | 878 | 15.14 | 0.000. |
|  | L gyrus frontalis medius | -40 46 20 | 339 | 12.94 | 0.000. |
|  | L middle cingulate gyrus | 0 -16 42 | 62 | 9.94 | 0.000. |
|  | R supramarginal gyrus | 56 -42 30 | 52 | 9,38 | 0.000. |
| **SMN** | L postcentral gyrus | -20 -32 70 | 6896 | 23.17 | 0.000. |
|  | R precentral | 52 -12 48 | 55 | 8.86 | 0.000. |
