# Supplementary material for: Frequency shifts in the anterior default mode network and the salience network in chronic pain disorder
Source: BMC Psychiatry. 2013 Mar 13;13:84. doi: 10.1186/1471-244X-13-84 (PMC3616999; doi:10.1186/1471-244X-13-84)
Supplement: Additional file 3: Table S2 — MNI-coordinates of the ICNs in the patient group. Results were thresholded at p = 0.05 and corrected for family wise error (FWE) on the voxel level with a cluster extent threshold of k= 50 voxels. [file 1471-244X-13-84-S3.doc]

**Table S2 MNI-coordinates of the ICNs in the patient group** Results were thresholded at p = 0.05 and corrected for family wise error (FWE) on the voxel level with a cluster extent threshold of k= 50 voxels.

| **Network** | **Region** | **MNI** | **k** | **T** | **p** |
| --- | --- | --- | --- | --- | --- |
| **aDMN** | R gyrus frontalis medius, pars orbitalis | 10 46 -4 | 3425 | 26.55 | 0.000 |
|  | L precuneus | -6 -56 22 | 277 | 10.29 | 0.000 |
| **pDMN** | R posterior cingulate cortex | 4 -42 24 | 3692 | 26.71 | 0.000 |
| **FIN** | L insula | -36 8 -2 | 1966 | 19.11 | 0.000 |
|  | L supplementary motor area | 0 8 44 | 1730 | 17.27 | 0.000 |
|  | R insula | 38 20 0 | 1064 | 16.64 | 0.000 |
|  | L gyrus frontalis medius | -34 44 30 | 435 | 11.54 | 0.000 |
|  | L supramarginal gyrus | -62 -44 28 | 125 | 10.59 | 0.000 |
|  | R gyrus frontalis medius | 36 48 30 | 123 | 8.76 | 0.000 |
| **SMN** | R gyrus parietalis superior | 22 -48 70 | 7101 | 26.42 | 0.000 |
|  |  |  |  |  |  |
