# Supplementary material for: Frequency shifts in the anterior default mode network and the salience network in chronic pain disorder
Source: BMC Psychiatry. 2013 Mar 13;13:84. doi: 10.1186/1471-244X-13-84 (PMC3616999; doi:10.1186/1471-244X-13-84)
Supplement: Additional file 4: Table S3 — MNI-coordinates of the group comparisons. Results were thresholded at p = 0.005, uncorrected at the voxel-level, and p < 0.05, corrected for multiple comparisons on the cluster level, with a cluster extent threshold of k = 50 voxels; p represents p on the voxel-level. [file 1471-244X-13-84-S4.doc]

| **Network** | **Region** | **MNI** | **k** | **T** | **p** |
| --- | --- | --- | --- | --- | --- |
| **aDMN: controls > patients** | L gyrus frontalis superior | -24 38 36 | 24 | 3.40 | 0.538 |
| **aDMN: patients > controls** | - | - | - | - | - |
| **pDMN: controls > patients** | - | - | - | - | - |
| **pDMN: patients > controls** | L cuneus | -10 -76 38 | 10 | 2.95 | 0.734 |
| **SMN: controls > patients** | R gyrus praecentralis | 52 -14 46 | 212 | 4.11 | 0.103 |
|  | L paracentral lobule | -14 -32 54 | 73 | 3.81 | 0.285 |
|  | R gyrus postcentralis | 18 -38 60 | 44 | 3.74 | 0.527 |
|  | L supplemental motor area | -10 2 70 | 10 | 3.21 | 0.928 |
| **SMN: patients > controls** | - | - | - | - | - |
| **FIN: controls > patients** | R gyrus frontalis inferior,  pars opercularis | 58 16 20 | 13 | 4.03 | 0.900 |
| **FIN: patients > controls** | L gyrus frontalis inferior,  pars opercularis | -44 10 10 | 69 | 3.82 | 0.308 |
|  | L middle cingulated cortex | -6 -22 42 | 11 | 3.47 | 0.921 |

**Table S3 MNI-coordinates of the group comparisons** Results were thresholded at p = 0.005, uncorrected at the voxel-level, and p < 0.05, corrected for multiple comparisons on the cluster level, with a cluster extent threshold of k = 50 voxels; p represents p on the voxel-level.
