# Supplementary material for: Frequency shifts in the anterior default mode network and the salience network in chronic pain disorder
Source: BMC Psychiatry. 2013 Mar 13;13:84. doi: 10.1186/1471-244X-13-84 (PMC3616999; doi:10.1186/1471-244X-13-84)
Supplement: Additional file 5: Table S4 — Correlation between functional connectivity and psychometric measurement. Results were thresholded at p < 0.005, uncorrected on the voxel-level, and p < 0.05, corrected on the cluster level, with a cluster extent threshold of k > 10 voxels; p represents p on the cluster level; R represents Pearson’s correlation-coefficient. No significant correlation was detected. [file 1471-244X-13-84-S5.doc]

**Table S4** **Correlation between functional connectivity and psychometric measurement** Results were thresholded at p < 0.005, uncorrected on the voxel-level, and p < 0.05, corrected on the cluster level, with a cluster extent threshold of k > 10 voxels; p represents p on the cluster level; R represents Pearson’s correlation-coefficient. No significant correlation was detected.

| ***BDI positive*** |  |  |  |  |  |  |
| --- | --- | --- | --- | --- | --- | --- |
| **Network** | **Region** | **MNI** | **k** | **T** | **p** | **R** |
| aDMN | L gyrus frontalis, pars orbitalis | -2 58 -6 | 15 | 3.26 | 0,396 | 0.5993 |
|  | L anterior cingulate cortex | -2 40 4 | 10 | 3.18 | 0.585 | 0.5894 |
| pDMN | - | - | - | - | - | - |
| SMN | R gyrus praecentralis | 24 -26 70 | 31 | 4.31 | 0.400 | 0.5737 |
|  | R middle cingulate cortex | 2 -16 50 | 50 | 3.82 | 0.6384 | 0.412 |
|  | L gyrus postcentralis | -22 -30 60 | 22 | 3.40 | 0.526 | 0.5035 |
|  | L paracentrale lobule | -6 -34 72 | 12 | 3.16 | 0.704 | 0.4809 |
| FIN | L gyrus frontalis medius | -26 48 26 | 21 | 3.79 | 0.455 | 0.5364 |
|  | R gyrus frontalis medius | 30 48 26 | 10 | 3.58 | 0.665 | 0.5151 |
| ***BDI negative*** |  |  |  |  |  |  |
| **Network** | **Region** | **MNI** | **k** | **T** | **p** | **R** |
| aDMN | L gyrus frontalis medialis | 0 54 16 | 93 | 3.86 | 0.32 | -0.7432 |
| pDMN | - | - | - | - | - | - |
| SMN | - | - | - | - | - | - |
| FIN | - | - | - | - | - | - |
|  |  |  |  |  |  |  |
| ***STAI-T positive*** |  |  |  |  |  |  |
| **Network** | **Region** | **MNI** | **k** | **T** | **p** | **R** |
| aDMN | - | - | - | - | - | - |
| pDMN | - | - | - | - | - | - |
| SMN | L precuneus | -14 -42 70 | 12 | 3.56 | 0.704 | 0.390 |
| FIN | - | - | - | - | - | - |
|  |  |  |  |  |  |  |
| ***STAI-T negative*** |  |  |  |  |  |  |
| **Network** | **Region** | **MNI** | **k** | **T** | **p** | **R** |
| aDMN | - | - | - | - | - | - |
| pDMN | - | - | - | - | - | - |
| SMN | - | - | - | - | - | - |
| FIN | L gyrus frontalis medialis | -6 16 42 | 22 | 3.85 | 0.439 | -0.5413 |
|  | L insula | -36 8 -6 | 22 | 3.70 | 0.439 | -0.5357 |
|  | L middle cingulate cortex | 0 8 40 | 13 | 3.45 | 0.600 | -0.5083 |
| ***BPI – item5 positive*** |  |  |  |  |  |  |
| **Network** | **Region** | **MNI** | **k** | **T** | **p** | **R** |
| N aDMN | - | - | - | - | - | - |
| pDMN | - | - | - | - | - | - |
| SMN | - | - | - | - | - | - |
| CIN | L gyrus frontalis medius | -34 44 22 | 41 | 3.95 | 0.221 | 0.6916 |
|  |  |  |  |  |  |  |
| ***BPI – item5 negative*** |  |  |  |  |  |  |
| **Network** | **Region** | **MNI** | **k** | **T** | **p** | **R** |
| N aDMN | R gyrus rectus | 4 52 -16 | 26 | 3.84 | 0.256 | -0.6812 |
| pDMN | L precuneus | -6 -64 36 | 16 | 3.59 | 0.376 | -0.6567 |
| SMN | - | - | - | - | - | - |
| CIN | - | - | - | - | - | - |
