# Supplementary material for: Frequency shifts in the anterior default mode network and the salience network in chronic pain disorder
Source: BMC Psychiatry. 2013 Mar 13;13:84. doi: 10.1186/1471-244X-13-84 (PMC3616999; doi:10.1186/1471-244X-13-84)
Supplement: Additional file 6: Table S5 — Pearson’s correlation between spectral power and psychometric measurements *The correlation with depression (BDI-I) is controlled for anxiety (STAI-T) and vice versa; the level of significance is p < 0.05; R represents the correlation-coefficient. No significant correlation was detected. [file 1471-244X-13-84-S6.doc]

**Table S5 Pearson’s correlation between spectral power and psychometric measurements** *The correlation with depression (BDI-I) is controlled for anxiety (STAI-T) and vice versa; the level of significance is p < 0.05; R represents the correlation-coefficient. No significant correlation was detected*.*

| **ICN** | **Psychometrics** | | **Spectral power at different frequency-bins in percent of the whole power** | | | | | |
| --- | --- | --- | --- | --- | --- | --- | --- | --- |
|  |  | | **0.0 – 0.04 Hz** | **0.04 – 0.08 Hz** | **0.08 – 0.12 Hz** | **0.12 – 0.16 Hz** | **0.16 – 0.20 Hz** | **0.20 – 0.24 Hz** |
| **aDMN** | **BPI** | **R** | 0.077 | -0.300 | -0.400 | -0.056 | 0.315 | 0.116 |
|  |  | **p** | 0.755 | 0.212 | 0.090 | 0.820 | 0.188 | 0.636 |
|  | **BDI-I*** | **R** | 0.188 | 0.040 | -0.232 | -0.268 | -0.436 | 0.186 |
|  |  | **p** | 0.427 | 0.866 | 0.325 | 0.252 | 0.055 | 0.433 |
|  | **STAI-T*** | **R** | 0.168 | 0.000 | 0.224 | 0.016 | 0.136 | -0.342 |
|  |  | **p** | 0.479 | 0.998 | 0.342 | 0.945 | 0.569 | 0.140 |
| **pDMN** | **BPI** | **R** | -0.445 | -0.150 | 0.284 | 0.415 | 0.381 | -0.044 |
|  |  | **p** | 0.056 | 0.540 | 0.238 | 0.077 | 0.108 | 0.859 |
|  | **BDI-I*** | **R** | -0.041 | 0.269 | -0.201 | -0.140 | -0.167 | 0.106 |
|  |  | **p** | 0.865 | 0.252 | 0.397 | 0.555 | 0.481 | 0.655 |
|  | **STAI-T*** | **R** | 0.105 | -0.258 | -0.004 | -0.90 | 0.087 | 0.090 |
|  |  | **p** | 0.661 | 0.272 | 0.987 | 0.706 | 0.717 | 0.706 |
| **FIN** | **BPI** | **R** | -0.105 | -0.090 | -0.293 | 0.188 | 0.227 | 0.103 |
|  |  | **p** | 0.669 | 0.714 | 0.224 | 0.441 | 0.350 | 0.674 |
|  | **BDI-I*** | **R** | 0.424 | -0.426 | -0.137 | -0.157 | -0.379 | 0.145 |
|  |  | **p** | 0.063 | 0.061 | 0.564 | 0.508 | 0.099 | 0.542 |
|  | **STAI-T*** | **R** | -0.020 | 0.208 | 0.078 | 0.014 | 0.338 | 0.325 |
|  |  | **p** | 0.932 | 0.379 | 0.745 | 0.954 | 0.145 | 0.162 |
| **SMN** | **BPI** | **R** | -0.301 | 0.272 | 0.267 | 0.445 | 0.044 | -0.197 |
|  |  | **p** | 0.210 | 0.261 | 0.269 | 0.056 | 0.858 | 0.419 |
|  | **BDI-I*** | **R** | 0.366 | 0.297 | -0.437 | -0.290 | -0.378 | -0.136 |
|  |  | **p** | 0.112 | 0.203 | 0.054 | 0.215 | 0.100 | 0.567 |
|  | **STAI-T*** | **R** | 0.031 | 0.007 | 0.076 | 0.002 | 0.075 | -0.134 |
|  |  | **p** | 0.898 | 0.976 | 0.749 | 0.992 | 0.753 | 0.572 |
